# Broadening the Heterologous Cross-Neutralizing Antibody Inducing Ability of Porcine Reproductive and Respiratory Syndrome Virus by Breeding the GP4 or M genes

**DOI:** 10.1371/journal.pone.0066645

**Published:** 2013-06-24

**Authors:** Lei Zhou, Yan-Yan Ni, Pablo Piñeyro, Caitlin M. Cossaboom, Sakthivel Subramaniam, Brenton J. Sanford, Barbara A. Dryman, Yao-Wei Huang, Xiang-Jin Meng

**Affiliations:** 1 Department of Biomedical Sciences and Pathobiology, College of Veterinary Medicine, Virginia Polytechnic Institute and State University (Virginia Tech), Blacksburg, VA, USA; 2 Key Laboratory of Animal Epidemiology and Zoonosis of Ministry of Agriculture, College of Veterinary Medicine and State Key Laboratory of Agribiotechnology, China Agricultural University, Beijing, People’s Republic of China; University of Georgia, United States of America

## Abstract

Porcine reproductive and respiratory syndrome virus (PRRSV) is one of the most economically important swine pathogens, which causes reproductive failure in sows and respiratory disease in piglets. A major hurdle to control PRRSV is the ineffectiveness of the current vaccines to confer protection against heterologous strains. Since both GP4 and M genes of PRRSV induce neutralizing antibodies, in this study we molecularly bred PRRSV through DNA shuffling of the GP4 and M genes, separately, from six genetically different strains of PRRSV in an attempt to identify chimeras with improved heterologous cross-neutralizing capability. The shuffled GP4 and M genes libraries were each cloned into the backbone of PRRSV strain VR2385 infectious clone pIR-VR2385-CA. Three GP4-shuffled chimeras and five M-shuffled chimeras, each representing sequences from all six parental strains, were selected and further characterized *in vitro* and in pigs. These eight chimeric viruses showed similar levels of replication with their backbone strain VR2385 both *in vitro* and *in vivo,* indicating that the DNA shuffling of GP4 and M genes did not significantly impair the replication ability of these chimeras. Cross-neutralization test revealed that the GP4-shuffled chimera GP4TS14 induced significantly higher cross-neutralizing antibodies against heterologous strains FL-12 and NADC20, and similarly that the M-shuffled chimera MTS57 also induced significantly higher levels of cross-neutralizing antibodies against heterologous strains MN184B and NADC20, when compared with their backbone parental strain VR2385 in infected pigs. The results suggest that DNA shuffling of the GP4 or M genes from different parental viruses can broaden the cross-neutralizing antibody-inducing ability of the chimeric viruses against heterologous PRRSV strains. The study has important implications for future development of a broadly protective vaccine against PRRSV.

## Introduction

Porcine reproductive and respiratory syndrome (PRRS), characterized by reproductive failure in sows and respiratory disease in piglets [1], is arguably the most economically important global swine disease in the past two decades [2]–[5]. Since its recognition in the United States in 1987 [6] and in Europe in 1990 [7], PRRS has devastated the global pork industry. According to a 2011 study, PRRS is estimated to cost the U.S. pork industry approximately $664 million per year, which is $104 million higher than the previous 2005 estimate [2]. In 2006, the emergence of highly pathogenic PRRS (HP-PRRS) in China and several Asian countries with 20–100% mortality nearly crippled the world’s biggest pork industry in China [8]–[10].

The causative pathogen of PRRS, porcine reproductive and respiratory syndrome virus (PRRSV), along with equine arteritis virus, lactate dehydrogenase-elevating virus of mice, and simian hemorrhagic fever virus [11], are classified in the family *Arteriviridae* of the order *Nidovirales* [11]. Two major genotypes of PRRSV have been identified: the European type (type 1) and the North American type (type 2) that share approximately 55–70% nucleotide sequence identity [7], [12]–[15]. Within the type 1 European PRRSV, there exist at least 3 distinct genetic lineages [16], and similarly, at least 9 distinct genetic lineages were identified within the type 2 North American PRRSV [17]. The extensive antigenic and genetic variations among field strains of PRRSV are largely responsible for the poor cross-protection of the current vaccines against heterologous strains [18]–[20]. The available modified live-attenuated vaccines (MLVs), which are all based on single PRRSV strains, can provide effective protection against homologous or genetically similar strains, but are not effective against the heterologous strains [21]–[23]. A biodegradable nanoparticle-entrapped vaccine was shown to induce cross-protective immunity against a heterologous PRRSV strain [24]. As the majority of field strains circulating in swine herds worldwide are genetically different from the MLVs, it is imperative to develop a second generation vaccine that can effectively protect against both homologous and heterologous strains [25]–[27].

The genome of PRRSV is a single-strand, positive-sense RNA molecule of approximately 15 kb with a 5′ cap and 3′ Poly(A) tail [28]–[30]. At least 9 open reading frames (ORFs) have been identified in the PRRSV genome: the ORF1a and ORF1b encode the replicase polyprotein responsible for viral replication and transcription, the ORFs2-4 encode virion-associated proteins GP2, GP2b, GP3, and GP4, respectively [31]–[33], and the remaining ORFs5-7 encode the major envelope (GP5), membrane (M), and nucleocapsid (N) proteins, respectively [34]–[36]. Also, a small ORF5a protein associated with PRRSV infection was recently reported [37], [38].

GP4 encoded by ORF4 is a minor structural protein of PRRSV with 178 amino acids (aa) and a molecular mass of approximately 31 kDa [39]. GP4 interacts with GP2 and GP3 to form a multi-protein complex important for viral infectivity [40]–[44]. It has been demonstrated that the GP4 mediates interglycoprotein interactions and together with GP2, serves as the viral attachment protein that binds CD163 during virus entry [45]. It has also been shown that GP4 is a GPI-anchored protein that co-localizes with the CD163 receptor in the lipid rafts and plays a role in the viral entry [46]. It is known that neutralizing antibody is directed to GP4 and the variable region in GP4 of type 1 European PRRSV induces neutralizing antibody against homologous but not heterologous virus strains [47]–[49]. The GP4-specific neutralizing antibody may drive PRRSV evolution [50].

The M protein encoded by ORF6 is an unglycosylated membrane protein of 18–19 kDa [28], [30], [34]. The M protein is important in virus assembly and budding [51] and is linked to GP5 as heterodimers via a disulfide bond in the N-terminal ectodomains [34], [52]. The M protein is a key target for PRRSV neutralization [53]. Co-expression of GP5 and M protein as heterodimers significantly improves the potency of PRRSV DNA vaccination [54]. Anti-M mAbs have been described, but the neutralizing epitopes in M gene have not yet been identified [53], [55].

DNA shuffling, known as molecular breeding, accelerates evolution of genes by mimicking the natural recombination process [56]–[62]. Compared with natural recombination, DNA shuffling can rapidly generate recombinants with new phenotypes in a very short period of time [56]. In the traditional DNA shuffling, the target gene fragments from selected parents are digested with DNase I to produce a pool of short DNA fragments, which are then reassembled by PCR to create a recombinant library. Desired properties of the shuffled genes such as broadened cross-neutralizing ability to heterologous virus strains can be screened and identified [60]. In our previous work, we have successfully attenuated PRRSV by shuffling its GP5 or GP5-M together [63]. We also shuffled the GP3 gene and identified a chimera that induced significantly higher levels of cross-neutralizing antibodies in pigs against a heterologous PRRSV strain [64].

Considering the important roles of GP4 and M proteins in PRRSV immune responses and neutralization, in this present study we molecularly bred PRRSV by traditional DNA shuffling of the GP4 or M genes from 6 heterologous PRRSV strains. Three GP4 gene-shuffled chimeric viruses and five M gene-shuffled chimeric viruses were selected for further *in vitro* and *in vivo* characterizations. The GP4 gene-shuffled chimera GP4TS14 and the M gene-shuffled chimera MTS57 were found to acquire significantly higher ability to elicit cross-neutralizing antibody against two heterologous strains of PRRSV in pigs.

## Materials and Methods

### Ethics Statement

The pig experiment in this study was approved by the Virginia Tech Institutional Animal Care and Use Committee (IACUC permit no. 10-124-CVM). All experimental procedures and animal care strictly follow the recommended guidelines by the American Veterinary Medical Association and the National Institutes of Health.

### Cells, Viruses and Viral Genes

**Figure 1:**
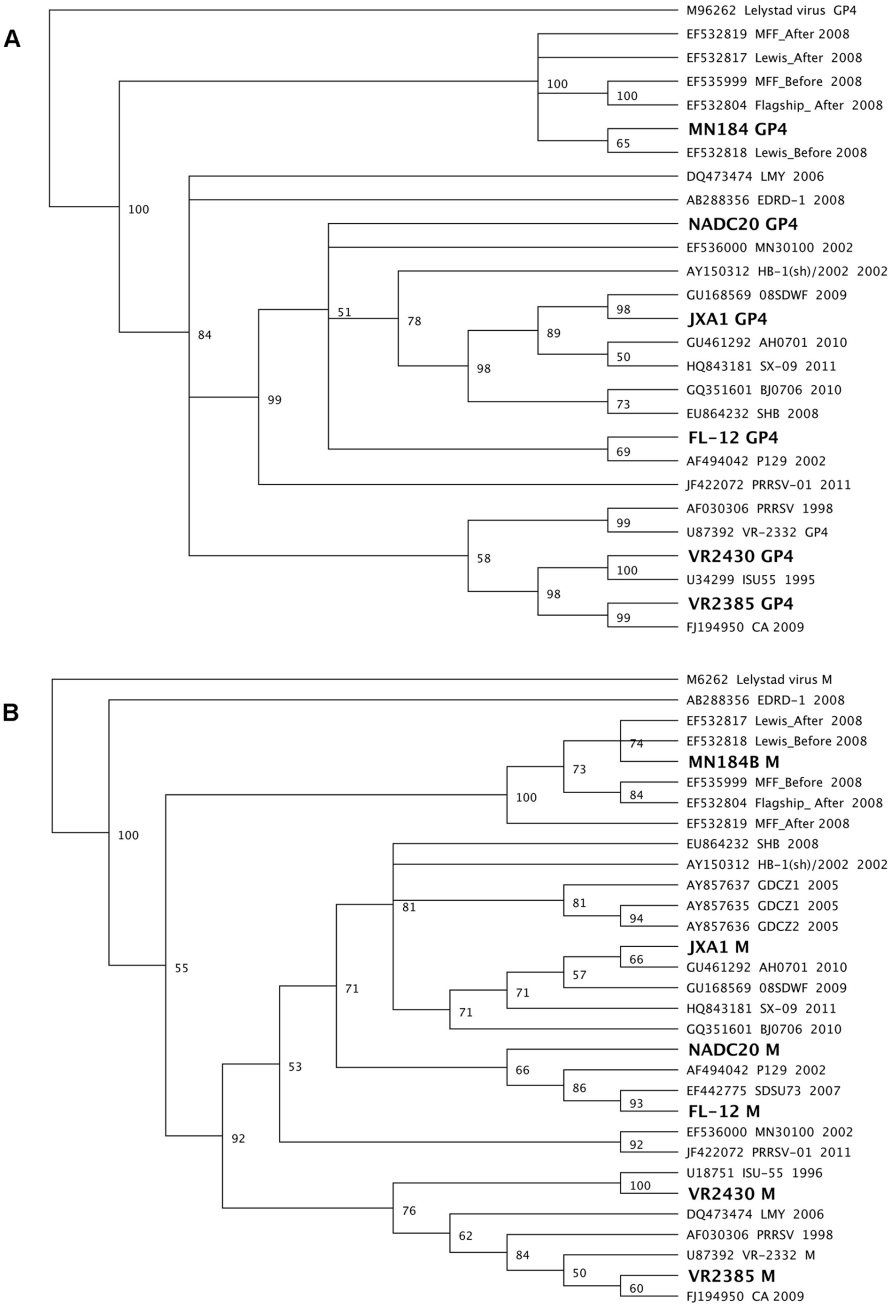
Two phylogenetic trees based on the sequence of GP4 (Panel A) or M (Panel B) genes of selected type 2 PRRSV strains. The six parental viruses (VR2385 JX044140, VR2430 JX050225, MN184B DQ176020, FL-12 AY545985, JXA1 EF112445, and NADC20 JX069953) used in the DNA shuffling are indicated with boldface in the trees. The phylogenetic trees were constructed using the neighbor-joining method with bootstraps of 100 replicates. The numbers above each branch indicate the bootstrap values (percentage of consensus support in bootstrap).

The BHK-21 and MARC-145 cells used for PRRSV rescue and propagation were cultured in DMEM with 10% FBS [65]. The type 2 PRRSV was classified into 9 distinct genetic lineages based on the GP5 gene sequences [66]. Six representative strains each from a different lineage or sublineage were selected for DNA shuffling in this study: MN184B (Accession no. DQ176020, lineage 1), VR2385 (Accession no. JX044140, lineage 5.1), VR2430 (Accession no. JX050225, lineage 5.2), Chinese highly pathogenic strain JXA1 (Accession no. EF112445, lineage 8.7), FL-12 (Accession no. AY545985, lineage 8.9), and NADC20 (Accession no. JX069953, lineage 9) [64]. Phylogenetic analyses based on the GP4 and M gene sequences of these six strains also confirmed that the six selected strains for this study are genetically distinct and separated into different lineages or sublineages (**Fig. 1**).

The GP4 and M genes of VR2385 and FL-12 strains were amplified from the full-length cDNA clones pIR-VR2385-CA [65] and pFL-12, respectively [67]. The GP4 and M genes of strain VR2430 were amplified by RT-PCR from the virus stock. The GP4 and M genes of strains MN184B, NADC20 and JXA1 were commercially synthesized (Genscript Inc).

### DNA Shuffling of the GP4 and M Genes, Respectively, from Six Genetically Distinct PRRSV Strains

For GP4 DNA shuffling, the DNA fragments of the GP4 genes from the six parental strains of PRRSV (**Fig. 1**) were equimolarly mixed with 1 µg DNA from each virus, and diluted to a 50 µl reaction in 50 mM Tris·HCl, pH 7.4 and 10 mM MgCl_2_. The DNA mixture was digested by 0.15 U of DNase I (Sigma) at 15°C for 2 min. The reaction was stopped by adding 5 µl of 0.5 M EDTA followed by 15 min incubation at 85°C for complete inactivation. DNA fragments with the approximate sizes of 50–150 bp were purified from 2% agarose gels using Qiaquick gel extraction kit (Qiagen). To reassemble the GP4 gene, the purified DNA fragments were subsequently added to the *Pfu* PCR mixture consisting of 1X *Pfu* buffer, 0.4 mM each dNTP, 0.06 U *Pfu* polymerase (Stratagene). A PCR program (95°C for 4 min; 35 cycles of 95°C for 30 s, 60°C for 30 s, 57°C for 30 s, 54°C for 30 s, 51°C for 30 s, 48°C for 30 s, 45°C for 30 s, 42°C for 30 s, 72°C for 2 min; and final incubation at 72°C for 7 min) without primers was performed to reassemble the digested DNA fragments. A 10 µl of the PCR products was run on an agarose gel to evaluate the efficiency of the reassembly. PRRSV-specific primer pair g4fu5F and g4fu3R (**Table 1**) were subsequently used to amplify the GP4 genes from the shuffled products. The PCR mixture contained 5 µl of reassembled products, 0.2 µM of each primer, 41 µl of PfuUltra II Hotstart PCR Master Mix (Stratagene). The PCR parameters included 4 min at 95°C; 25 cycles of 30 s at 95°C, 30 s at 55°C, 45 s at 72°C; a final 7 min at 72°C. After amplification, a single band of the shuffled products with an expected size of 576 bp was purified from 2% agarose gels.

**Table 1:** Oligonucleotide primers used in this study.

Primers*	Sequence (5′-3′)	Purpose
g4fu5F	GGCAATTGGTTTCACCTAGAATGGCTGCGYCCYTTCTT	Shuffled region
g4fu3R	CCCCAACATACTTAAACATTYAAATKGCCARYANGRATGG	Shuffled region
EcoRVF	GGAGTTCTTGGTGTCCATTGTTG	Flanking fragment
g4fu5R	TCTAGGTGAAACCAATTGCC	Flanking fragment
g4fu3F	GAATGTTTAAGTATGTTGGGG	Flanking fragment
Rluc-7R	GGACAAACCACAACTAGAATGCAGTG	Flanking fragment
g3fu3F	GGTCGACGGCGGCAATTGGTTTC	GP4 sequencing
Acl I 5R	CACATAGCGTCAAGTTGTAAATCA	GP4 sequencing
mfu5F	CGGAACAATGGGGTCGTCCTTAG	Shuffled region
mTS3R	ACAGCTTTTCTGCCACCCAACACG	Shuffled region
mfu5R	CTAAGGACGACCCCATTGTTCCG	Flanking fragment
mTS3F	GTGTTGGGTGGCAGAAAAGCTGTYAARCAGGGAGTGGTAAACCTYGTYAAATATGCCAAATAA	Flanking fragment
mseqF	CGTTGGCGGTCGCCTGTCATC	M sequencing
mseqR	GCCGCTCACTAGGGGTAAAGTG	M sequencing

* Primer orientations: F, Forward; R, Reverse.

The process of DNA shuffling of the M gene from 6 different strains of PRRSV was essentially the same as that of GP4 gene shuffling described above. The PRRSV-specific primers used for amplifying the M genes after reassembly were mfu5F and mTS3R (**Table 1**). After amplification, a single band of the M gene shuffled products with an expected size of 492 bp was visualized and purified from 2% agarose gels.

### Construction of Chimeric PRRSV cDNA Clone Libraries with Shuffled GP4 and M Genes Respectively

The DNA-launched PRRSV infectious clone pIR-VR2385-CA [65] was used as the backbone to clone the shuffled GP4 gene fragments and the shuffled M gene fragments, respectively. To insert the shuffled GP4 gene fragments into the full-length infectious clone plasmid DNA utilizing the unique restriction sites Bsr GI and Xbal I, we amplified two flanking fragments respectively with two pairs of primers (**Table 1**), EcoRVF+g4fu5R and g4fu3F+Rluc-7R, using the pIR-VR2385-CA plasmid DNA as the template. Subsequently, a fusion PCR was used to link the shuffled GP4 gene fragment with the two flanking fragments. The fusion product was then cloned into the genomic backbone pIR-VR2385-CA infectious clone to construct the chimeric PRRSV libraries. The recombination efficiency of GP4 shuffling was analyzed by sequencing the GP4 region with primer g3fu3F (**Table 1**) from 45 randomly selected clones. The nucleotide sequences of the shuffled GP4 regions were compared with those of the six parental strains by clustalW method. The mutated nucleotide sites different from the parental strains serve as the markers to delineate regions of crossover. If the sequence is unique to a particular parental strain between two crossover sites, then that region between the two crossovers is derived from that particular parental strain. The clones containing chimeric sequences representing all six parental strains were identified.

Similarly, for constructing chimeric PRRSV libraries containing the shuffled M genes, the primers Vg4fu5F+mfu5R and mTS3F+Rluc-7R were used to amplify the two flanking fragments of the shuffled M genes regions using the pIR-VR2385-CA infectious clone plasmid DNA as the template. The two flanking fragments were then fused to the shuffled M genes by a fusion PCR. The fusion products containing the shuffled M gene were subsequently cloned into the genomic backbone of the pIR-VR2385-CA infectious clone by using restriction enzyme sites Acl I and Xba I to construct the chimeric PRRSV libraries. A total of 65 clones were randomly selected and sequenced using primers mseqF and mseqR (**Table 1**). Sequence analyses identified well-shuffled clones containing sequences from all six parental strains of PRRSV.

### Chimeric Virus Rescue and Identification

To rescue the shuffled chimeric viruses, eight well-shuffled chimeric clones (three clones from GP4 gene shuffled library and five clones from M gene shuffled library) were transfected into the BHK-21 cells, respectively, as described previously [65]. At 24 h post-transfection, the supernatant of the transfected cells was harvested and subsequently passaged onto MARC-145 cells. Immunofluorescence assay (IFA) was used to identify the rescued viruses by using anti-PRRSV N monoclonal antibody SDOW17 (Rural Technologies, Inc) [68]–[70]. The GP4 and M gene regions from the rescued chimeric viruses were amplified and sequenced to confirm that the chimeric viruses were successfully rescued.

### *In vitro* Growth Characterization of the Shuffled Chimeric Viruses

To analyze if the chimeric viruses with the shuffled GP4 or M genes alter their growth characteristics *in vitro*, MARC-145 cells seeded in 96-well plates as confluent monolayers, were infected with the parental backbone strain VR2385 and chimeric viruses containing the shuffled GP4 or M genes at the same multiplicity of infection (m.o.i.) of 0.1, respectively. The infectious titers of the viruses harvested at 6, 12, 24, 36, 48, 60, 72 and 84 hours post-infection (hpi) were determined by IFA in MARC-145 cells and quantified as 50% tissue culture infective dose per ml (TCID_50_/ml). Triple independent repeats were performed in this study.

### An Animal Study to Characterize the Chimeric Viruses *in vivo*

To further analyze the growth characteristics of these eight chimeric viruses *in vivo* and to evaluate their ability to induce neutralizing and cross-neutralizing antibodies in pigs, a total of 32 specific-pathogen-free (SPF) pigs of 4 weeks of age that were negative for PRRSV were divided into 8 groups of 3 pigs per group for each of the 8 chimeric viruses (groups 1–8), and the remaining 8 pigs were assigned as the positive and negative control groups with 4 pigs per group. Pigs in groups 1–8 were each inoculated with 2 ml of each of eight different chimeric viruses intramuscularly (2×10^5^ TCID_50_/pig). Pigs in the positive control group were intramuscularly inoculated with 2 ml of the parental VR2385 virus (2×10^5^ TCID_50_/pig) and the pigs in the negative control group were mock-infected with 2 ml cell culture media. Serum samples from each pig were collected prior to inoculation and weekly thereafter, and all pigs were euthanized at 43 days post-inoculation (DPI).

### Characterization of the Replication Kinetics and Antibody Responses of Chimeric Viruses in Pigs

The infectious titers of the chimeric viruses in serum samples of infected pigs were determined by IFA in MARC-145 cells as previously described [64]. Briefly, serum samples were 10-fold-serially-diluted (10^0^, 10^−1^ to 10^−6^; 50 µl/well) to inoculate MARC-145 cells. After incubation for 20 hours at 37°C, the cells were stained with anti-PRRSV antibody SDOW17 [68]–[70] and FITC-conjugated goat anti-mouse IgG (KPL,Inc) to determine the viral titers. The viral titers in serum samples collected at 7 and 14 DPI were quantified as TCID_50_/ml.

Additionally, the GP4 or M gene shuffled regions of chimeric viruses recovered from pigs at 14 DPI were amplified by RT-PCR using primers g3fu3F+Acl I 5R for GP4 and mseqF+mseqR for M (**Table 1**) with the following parameters: 95°C for 4 min; 40 cycles of 95°C for 30 s, 55°C for 60 s, 72°C for 90 s; and a final incubation at 72°C for 7 min. The PCR products were purified and sequenced. The IDEXX HerdCheck X3® ELISA for PRRS was used to detect the anti-PRRSV antibody level in serum samples (Iowa State University Diagnostic Laboratories, Ames, Iowa).

### Serum Virus Neutralization (SVN) Assay

To detect the neutralizing and cross-neutralizing antibody-inducing ability of each chimeric virus to the homologous (VR2385) and heterologous strains of PRRSV (VR2430, MN184B, FL-12 and NADC20), respectively, the neutralizing antibody (NA) titers against different virus strains were determined at 43 DPI. Briefly, serum samples were serially 2-fold diluted in DMEM media supplemented with 2% FBS. The diluted serum samples, starting at 1∶2 dilutions, were mixed with an equal volume of the respective virus at a titer of 2×10^3^ TCID_50_/ml and incubated for 1 h at 37°C. The mixtures were then added to a 96-well cell culture plate with confluent monolayers of MARC-145 cells and incubated for another 1 h at 37°C. After removing the inocula and washing the cells twice with PBS, 50 µl of fresh DMEM media supplemented with 2% FBS was added to each well. After incubation for 20 h at 37°C, the cells were fixed with 80% acetone (Sigma) and stained with an anti-PRRSV antibody SDOW17 and FITC-conjugated goat anti-mouse IgG [68]–[70] by IFA to detect evidence of virus replication. The neutralizing antibody titers were expressed as the highest dilution that showed a 90% or greater reduction in the numbers of fluorescent foci compared to that of the serum samples from the negative control group in the same dilution as described previously [33]. Three independent tests were performed for each serum sample, and the average neutralizing antibody (NA) titers of each shuffled virus group (average of three pigs) were calculated and compared with that of the backbone parental VR2385 group (average of four pigs) by unpaired *t* test, with 95% confidence level.

To further assess the kinetics of neutralizing antibodies, the NA titers in serum samples collected at 28, 35 and 43 DPI from pigs infected with chimeras GP4TS14 and MTS57 were tested and compared with that of the backbone parental virus VR2385.

### Sequence Alignment of the GP4 and M Gene Regions of the Chimeric Viruses

To analyze the potential correlation between amino acid mutation and improved cross-neutralizing ability of the chimeras in infected pigs, the GP4 and M amino acid sequences of the chimeric viruses and their six parental viruses were aligned and analyzed by clustalW method.

### Statistical Analyses

The differences between the samples from the chimeric virus groups and samples from parental strain VR2385 group were evaluated by unpaired Student’s *t* test, with 95% confidence level.

## Results

### Generation of a GP4 Gene-shuffled PRRSV Library

The GP4 gene fragments from each of the six parental PRRSV strains were digested by DNase I and reassembled by PCR without primer. A PCR product with the expected size of 576 bp was amplified with PRRSV-specific primers flanking the shuffled GP4 region using the reassembly products as the template. An additional round of DNA shuffling process was iterated as described previously [71], [72] by using the shuffled DNA pool as the parents to produce a well-shuffled library of the GP4 genes.

To insert the shuffled GP4 fragments into the backbone PRRSV infectious clone pIR-VR2385-CA, two flanking fragments (1104 bp and 1957 bp, respectively) amplified from the pIR-VR2385-CA plasmid DNA were linked to the shuffled GP4 fragments by a fusion PCR. The unique restriction enzyme sites Bsr GI and Xba I present in the flanking fragments were used to clone the fusion product into the backbone PRRSV infectious clone plasmid pIR-VR2385-CA to produce the GP4 shuffled PRRSV infectious cDNA library.

### Generation of an M Gene-shuffled PRRSV Library

Similar to the GP4 gene shuffling, the shuffled M gene fragments were linked with two flanking fragments (950 bp and 896 bp, respectively) by a fusion PCR. The unique restriction enzyme sites Acl I and Xba I present in the flanking fragments were used to insert the shuffled M gene fragments into pIR-VR2385-CA plasmid DNA clone to produce the M gene-shuffled PRRSV library.

### Generation of Infectious Chimeric Viruses Containing Shuffled GP4 or M Genes from 6 Different PRRSV Strains by Traditional DNA Shuffling

**Figure 2:**
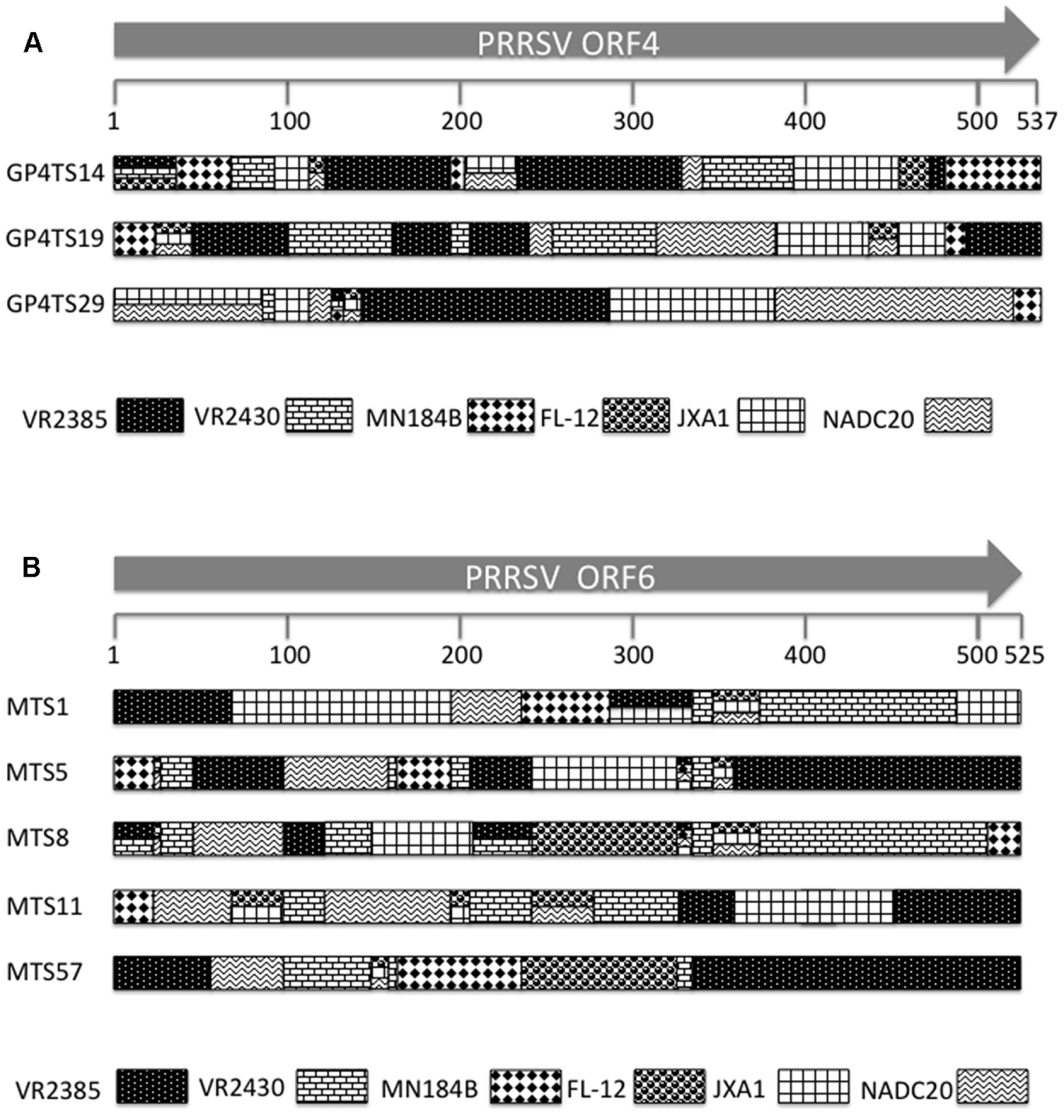
Schematic diagrams of the GP4 (Panel A) or M (Panel B) genes sequences in eight infectious chimeras (GP4TS14, GP4TS19, GP4TS29, MTS1, MTS5, MTS8, MTS11 and MTS57) generated by DNA shuffling. Each pattern in the shuffled genes represents one of the six different individual parental virus strains, which are shown at the bottom. The crossovers are delineated by the diversity of nucleotides because of the incorporation of the sequences from different parental virus strains. Multiple patterns that are displayed at the same region of the shuffled genes indicate that the sequence in this particular region was conserved at the corresponding parental virus strains. The numbers under the arrows indicate the nucleotide position.

**Figure 3:**
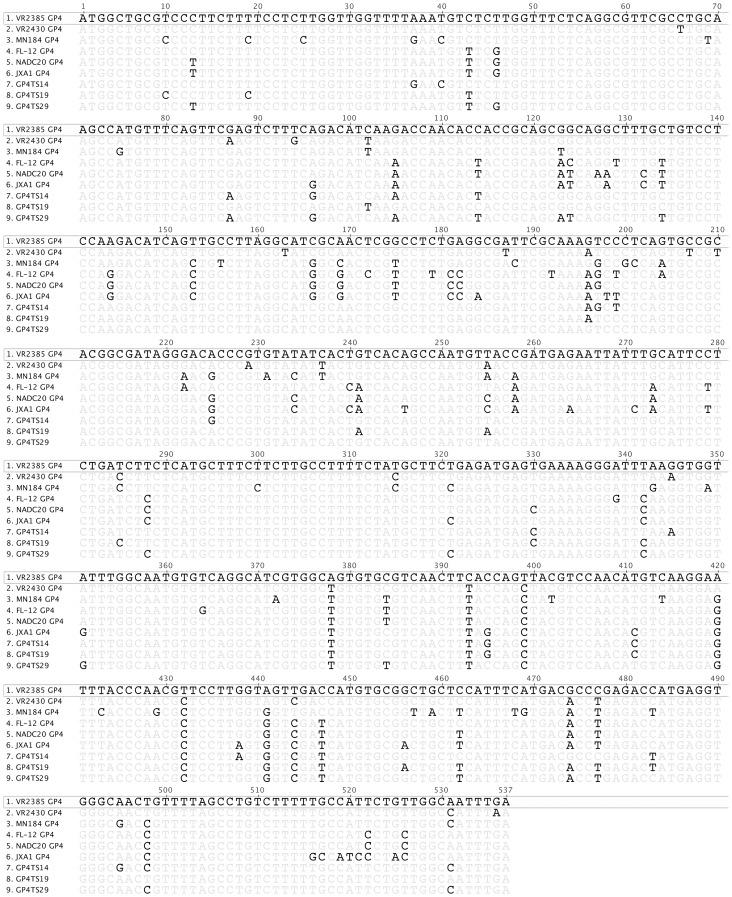
Nucleotide sequence alignment of the three GP4-shuffled chimeric viruses and their parents by clustalW method. The sequence of the backbone virus VR2385 was shown on top, and only differences were indicated for other viruses.

**Figure 4:**
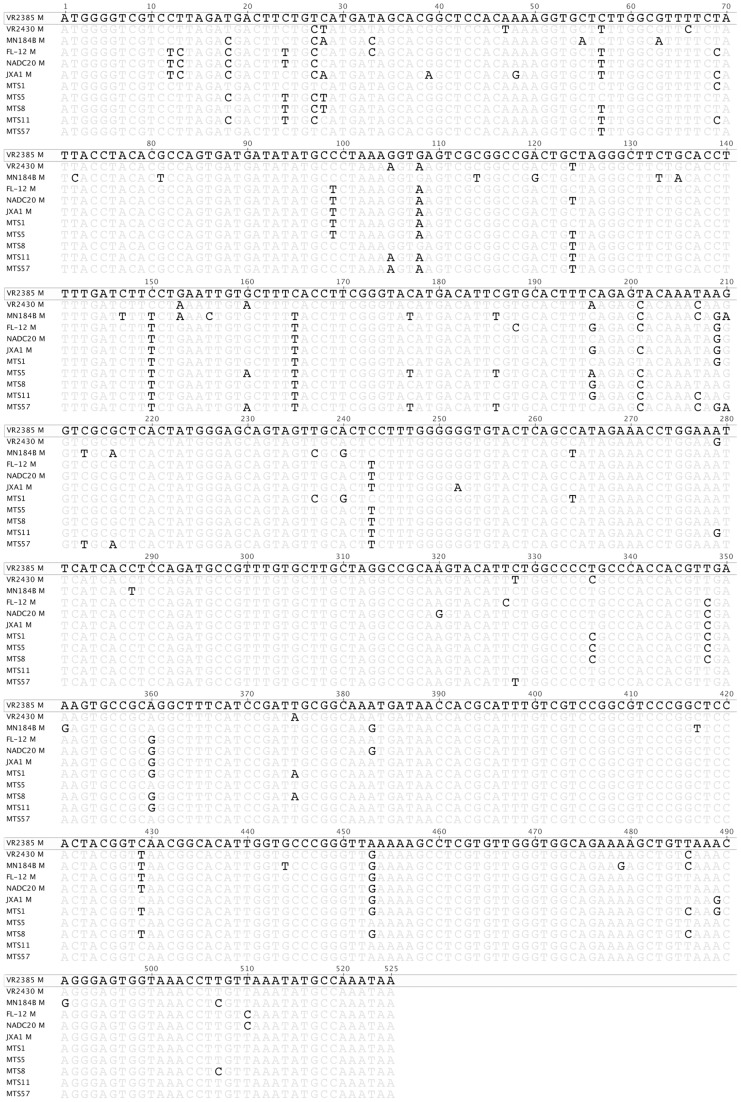
Nucleotide sequence alignment of the five M-shuffled chimeric viruses and their parents by clustalW method. The sequence of the backbone virus VR2385 was shown on top, and only differences were indicated for other viruses.

**Figure 5:**
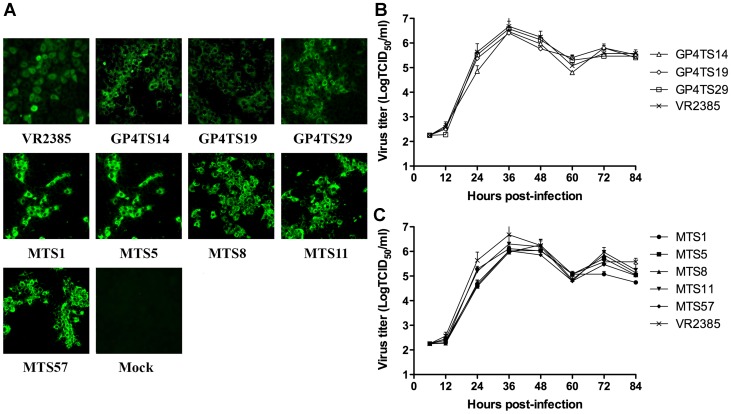
Rescue of infectious chimeric viruses from shuffled infectious clones and *in vitro* growth kinetics of the shuffled chimeric viruses. Immunofluorescence assay (IFA) (**Panel A**) was performed with anti-PRRSV N protein monoclonal antibody (SDOW17) to confirm that the chimeric viruses were successfully rescued in MARC-145 cells infected with the supernatant of BHK-21 cells transfected with eight individual clones generated by DNA shuffling (GP4TS14, GP4TS19, GP4TS29, MTS1, MTS5, MTS8, MTS11 and MTS57). Parental backbone strain VR2385 and mock infection were included as positive and negative controls, respectively. The growth kinetics of GP4-shuffled chimeric viruses (**Panel B**) and M-shuffled chimeric viruses (**Panel C**) in MARC-145 cells were determined by measuring the infectious viral titers (TCID_50_/ml) at indicated time points post-infection using the microtitration infectivity assay. The experiments were done in triplicate, and the bars indicated standard errors.

Randomly selected individual clones including 45 clones from the GP4-shuffled library and 65 clones from M-shuffled library were each sequenced for the respective shuffled region. Based on sequence analyses and comparison with the six parental virus strains, three chimeric clones from GP4 shuffled library, designated as GP4TS14, GP4TS19 and GP4TS29, and five chimeric clones from M shuffled library, designated as MTS1, MTS5, MTS8, MTS11 and MTS57, were all identified to contain sequences from all six parental virus strains (**Fig. 2**). These 8 chimeric viruses were selected for further *in vitro* and *in vivo* characterization. The GP4 and M gene nucleotide sequences of the chimeric viruses were compared to their six corresponding parental viruses respectively (**Fig. 3** **and** **Fig. 4**). The eight chimeric viruses were successfully rescued from MARC-145 cells that were infected with the supernatant of BHK-21 cells transfected with DNA-launched PRRSV infectious clone containing the shuffled GP4 or M genes (**Fig. 5A**).

### The shuffled chimeric viruses had similar levels of replication *in vitro* with the backbone VR2385 virus

To determine if GP4 or M gene shuffling has any effect on the replication ability of these eight chimeric viruses *in vitro*, we determined and compared the growth kinetics of the chimeric viruses with that of the backbone VR2385 virus in MARC-145 cells. The results indicated the replication levels of GP4-shuffled or M-shuffled chimeric viruses were not significantly different from that of the backbone VR2385 virus (**Fig. 5B and 5C**), suggesting that the GP4 and M genes shuffling did not significantly impair the replication ability of these chimeric viruses in MARC-145 cells.

### The Shuffled Chimeric Viruses were Similar in General in their Infection Dynamics to the Backbone VR2385 Virus in Experimentally-infected Pigs

**Figure 6:**
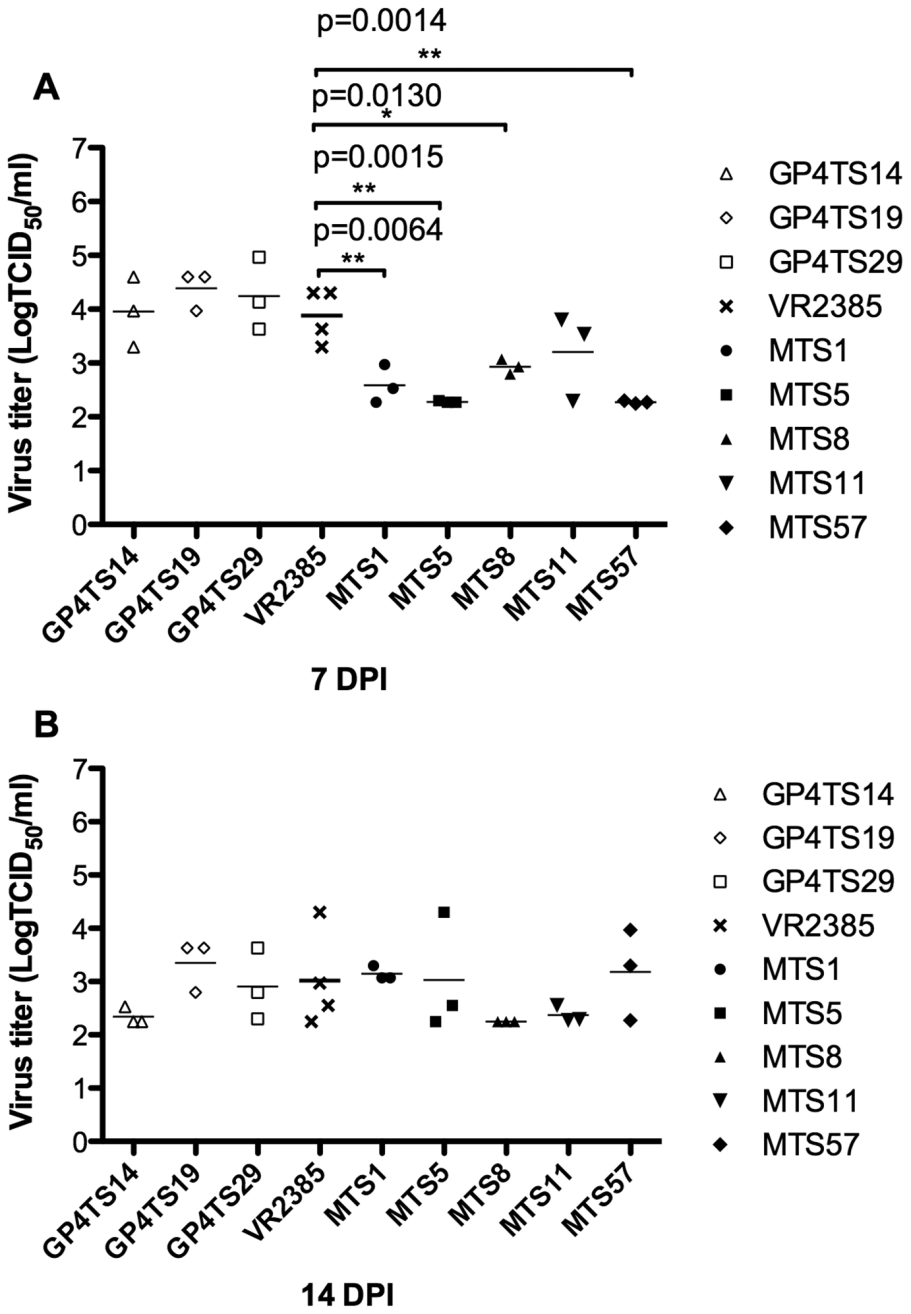
Infectious virus titers in sera of pigs experimentally-infected with eight chimeric viruses, with the backbone virus VR2385 or with cell culture media at 7 (Panel A) and 14 (Panel B) days post-inoculation (DPI). The asterisk (*) sign indicated a significant difference between the chimeric virus and backbone strain VR2385.

Pigs in the eight test groups 1–8 (3 pigs/group) were inoculated with each of the 8 shuffled chimeric viruses, whereas the positive and negative controls groups (4 pigs/group) were inoculated with the backbone VR2385 virus and DMEM media, respectively. The infectious titer of viruses in serum samples were determined by IFA in MARC-145 cells at 7 and 14 DPI. There was no significant difference between the titers of GP4-shuffled chimeric viruses and that of VR2385 in serum samples collected at both 7 and 14 DPI. The infectious virus titers of the M-shuffled chimeric viruses (MTS1, MTS5, MTS8 and MTS57) were significantly lower than that of backbone VR2385 virus at 7 DPI (*p* = 0.0064, *p* = 0.0015, *p* = 0.0130 and *p* = 0.0014, respectively) (**Fig. 6A**). However, there was no significant difference among the groups at 14 DPI (**Fig. 6B**). The viremia was cleared at 21 DPI for the GP4TS14 and GP4TS29 groups, and at 28 DPI for the other groups. The results of the infection dynamics and kinetics suggested the DNA shuffling of GP4 or M gene did not significantly impair the viral replication *in vivo*, even though the infectious titers of the M gene-shuffled chimeric viruses were temporarily lower than that of the backbone VR2385 virus at 7 DPI.

### The Chimeric Viruses Elicited Similar Levels of Anti-PRRSV Antibody Responses Compared to the Backbone VR2385 Virus in Pigs

The anti-PRRSV antibody responses in weekly serum samples were tested by the IDEXX HerdCheck X3® ELISA kit and displayed as sample-to-positive (S/P) ratio. Samples were considered positive if the calculated S/P ratio was equal to 0.4 or greater. The results showed that pigs in all inoculated groups seroconverted at 14 DPI, and remained seropositive for the duration of the study. The eight chimeric viruses induced similar levels of anti-PRRSV antibody responses in pigs (data not shown). When compared to the antibody titer (S/P ratio) of the backbone strain VR2385-inoculated pigs, there was no significant difference between shuffled chimeric virus groups and the VR2385 group at any time point. The four pigs in the negative control group were seronegative for anti-PRRSV throughout the study.

### DNA Shuffling of GP4 or M Gene Broadens the Cross-neutralizing Antibody-inducing Capability of Respective Chimeric Viruses Against Heterologous Strains of PRRSV

**Figure 7:**
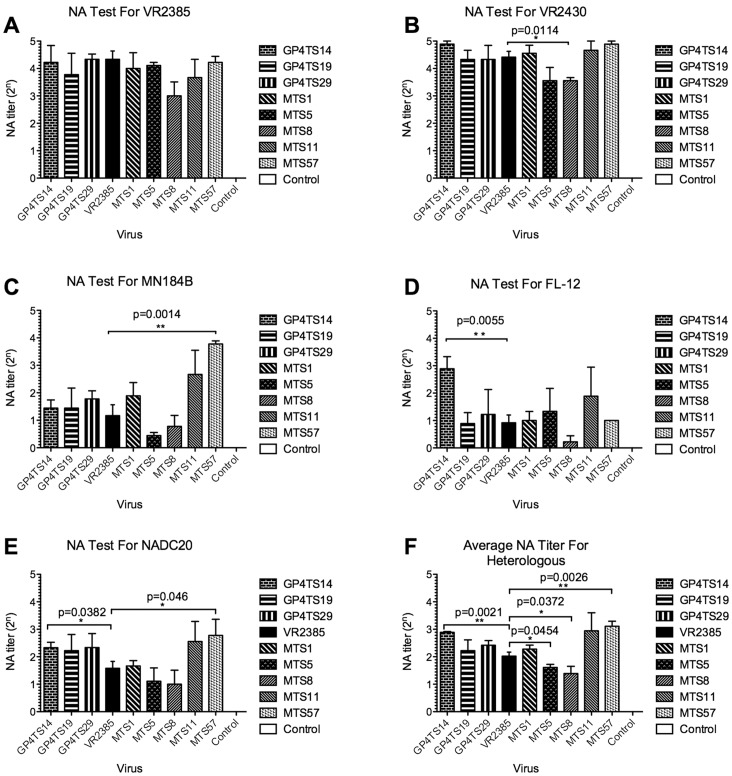
Neutralizing antibody titers against homologous and heterologous strains of PRRSV in serum samples of pigs experimentally infected with eight chimeric viruses or with the backbone virus VR2385, respectively: (A-E): Neutralizing antibody (NA) titers in serum samples collected at 43 DPI from pigs infected with indicated chimeric viruses or VR2385. *In vitro* cross-neutralization SVN test of respective serum samples was performed against the five available parental virus strains used in the DNA shuffling including VR2385, VR2430, MN184B, FL-12 and NADC20, respectively. **F**: The average NA titers against all four heterologous strains (VR2430, MN184, FL-12 and NADC20). The NA titers against the heterologous strains in pigs infected with chimeric viruses were averaged and summarized individually, and compared with that in pigs infected with VR2385. The NA titers were calculated as the highest 2-fold dilution (2^n^) of the serum sample that showed a 90% or greater reduction in the number of positive fluorescent foci, compared to that of the serum samples from the negative control group in the same dilution. Three independent experiments were performed for each test, and the average NA titer for each group of pigs (3 pigs in test groups and 4 pigs in control groups) was shown in the figure. The error bars indicate standard errors. The asterisk (*) signs indicate a significant difference between the group of chimera pigs and the backbone parental strain VR2385. One asterisk (*) signs indicate *p*<0.05 and two asterisk (**) signs indicate *p*<0.01. The *p* values are shown above the asterisk signs.

To investigate if the DNA shuffling of GP4 or M gene from different strains can produce chimeric viruses with an broadened cross-neutralizing antibody-inducing ability against heterologous strains, we determined and compared the anti-PRRSV specific neutralizing antibody titers in sera collected at 43 DPI by a cross-neutralization serum SVN assay with each of the five available parental virus strains (VR2385, VR2430, MN184B, FL-12 and NADC20), respectively. The NA titers were expressed as highest times (“n”) of 2-fold serial dilution (2^n^ or the power of 2) of the serum sample that showed a 90% or greater reduction in the number of positive fluorescent foci. In the SVN assay against the backbone VR2385 virus, the average NA titer of serum samples from positive control group was 4.33 (1∶20), and the NA titer for the chimeric virus groups were between 3 (1∶8) to 4.22 (1∶19) (**Fig. 7A**), which were not significantly different from that of the backbone VR2385 virus, suggesting that the GP4 or M gene shuffling did not impair the neutralizing antibody-inducing ability of the chimeric viruses against their respective backbone parental strain.

In the SVN test against strain VR2430, which belongs to the same lineage but a different sublineage with the backbone virus VR2385 in the phylogenetic tree [66], the NA titers of chimeric viruses GP4TS14 (4.89, 1∶30), MTS1 (4.56, 1∶24), MTS11 (4.67, 1∶25) and MTS57 (4.83, 1∶28) were higher than that of the VR2385 group (4.42, 1∶21), although the difference between chimeric viruses and backbone VR2385 virus is not statistically significant. However, the NA titers of chimeras MTS5 (3.56, 1∶12) and MTS8 (3.56, 1∶12) groups were lower than that of VR2385, and there was a significant difference between the chimera MTS8 group and VR2385 group (*p* = 0.0114). The NA titers of the remaining GP4TS19 and GP4TS29 groups were similar with that of VR2385 group (**Fig. 7B**).

In the SVN test against the heterologous strain MN184B, the NA titers of all inoculated groups, which were between 0.44 (1∶1) and 3.83 (1∶14), were lower than that against homologous backbone strain VR2385. With the exception of chimeras MTS5 and MTS8, the NA titers of the chimeric viruses groups were equal or higher than that of backbone virus VR2385 group (1.17, 1∶2) with the NA titer of the chimera MTS57 group (3.83, 1∶14) nearly 7 times higher than that of the VR2385 group (*p* = 0.0014) (**Fig. 7C**). The results of SVN test against the strain FL-12 showed that the NA titer of chimera GP4TS14 group (2.89, 1∶7) was significantly higher (*p* = 0.0055) than that of the backbone strain VR2385 group (0.91, 1∶2). Similarly, the NA titer of the chimera MTS11 group was higher than that of VR2385, although the difference was not significant (**Fig. 7D**). Importantly, the results of SVN test against NADC20 virus revealed that the chimeric viruses GP4TS14 and MTS57, which induced higher cross-neutralizing antibody titers against MN184B and FL-12, also induce significantly higher NA titers against NADC20 (*p* = 0.0382 and *p* = 0.046 respectively) (**Fig. 7E**), further indicating that the DNA shuffling of GP4 or M gene produced chimeric viruses with a broader cross-neutralizing antibody-inducing activity against multiple heterologous PRRSV strains.

To further analyze the overall cross-neutralizing antibody-inducing ability of the chimeric viruses, the average NA titers against four heterologous strains (VR2430, MN184B, FL-12 and NADC20) were composited and compared with that of the backbone VR2385 virus group (**Fig. 7F**). The average NA titers of the chimeras GP4TS14 and MTS57 groups were significantly higher than that of the VR2385 group (*p* = 0.0021 and *p* = 0.0026, respectively), and similarly, the average NA titer of the chimera MTS11 group was also higher than that of VR2385 group, but the difference between these two was not statistically significant. The average NA titers of MTS5 and MTS8 groups against heterologous strains were significantly lower than that of VR2385 (*p* = 0.0454 and *p* = 0.0372 respectively). The other chimeric strains elicited a similar level of cross-neutralizing antibodies, as did VR2385. The neutralizing antibodies in the serum samples of negative control group against each parental virus strain were undetectable. The composite results of cross-neutralizing SVN test further indicated that DNA shuffling of GP4 or M gene from different strains contributed to a broadened cross-neutralizing antibody-inducing ability of chimeric viruses GP4TS14 and MTS57.

### Kinetics of the Appearance of Neutralizing and Cross-neutralizing Antibodies in Experimentally Infected Pigs

**Figure 8:**
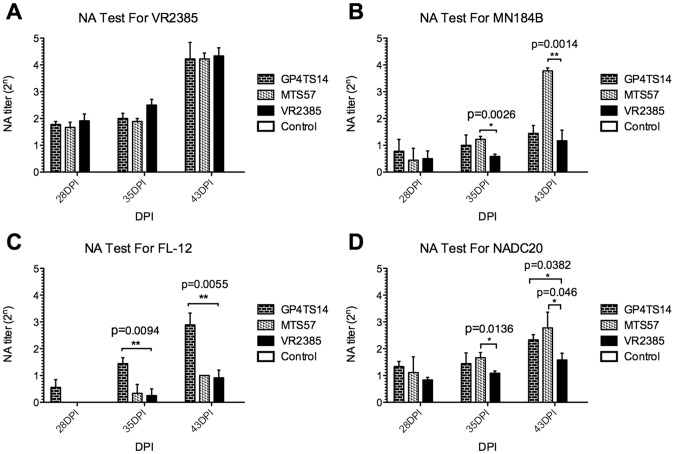
Kinetics of neutralizing antibodies in serum samples collected at 28, 35 and 43 days post-inoculation (DPI) from pigs experimentally infected with chimeric viruses GP4TS14 and MTS57 or with the backbone virus VR2385, respectively. The NA titers in serum samples at 28, 35 and 43 DPI from pigs experimentally infected with chimeras GP4TS14 and MTS57 or with VR2385 were examined by an *in vitro* serum virus neutralization test against four parental virus strains VR2385, MN184B, FL-12 and NADC20, respectively (**A–E**). Each test was performed in triplicate, the average NA titers of 3 or 4 pigs in each group are shown in the figure and the bars indicate standard errors. The asterisk (*) signs indicate a significant difference between the chimeric virus and the parental strain VR2385. One asterisk (*) signs indicate *p*<0.05 and two asterisk (**) signs indicate *p*<0.01. The *p* values are shown above the asterisk signs.

To further evaluate the dynamics of neutralizing antibodies at different time points post-inoculation, the NA titers against the backbone homologous (VR2385) and three heterologous parental strains (MN184B, FL-12, and NADC20) of the chimeras GP4TS14 and MTS57 groups, which both acquired an improved cross-neutralizing antibody inducing ability by DNA shuffling, were compared with the NA titers of the backbone virus VR2385 at 28, 35 and 43 DPI. In the SVN test against VR2385, the NA titers of the two chimeric groups and VR2385 group were similar at all 3 time points, ranging from 1.92 (1∶4) at 28 DPI to 4.33 (1∶20) at 43 DPI (**Fig. 8A**). In the SVN test against heterologous MN184B, the NA titers of the three groups were all less than 1(1∶2) at 28 DPI. At 35 DPI, the NA titers of the two chimeric groups were higher than that of the VR2385 group with a significant difference between MTS57 and VR2385 groups (*p* = 0.0026). At 43 DPI, only the NA titer of chimera MTS57 group was significantly higher than that of the VR2385 (*p* = 0.0014) (**Fig. 8B**). The NA titers against FL-12 were undetectable until 35 DPI in groups MTS57 and VR2385. The NA titers of GP4TS14 group were significantly higher than that of VR2385 both at 35 and 43 DPI (*p* = 0.0094 and *p* = 0.0055, respectively) (**Fig. 8C**). When the NA titers against NADC20 were compared among these three groups, the results showed that the NA titers of chimeras GP4TS14 and MTS57 groups were higher than that of VR2385 group at all three time points with a significant difference between MTS57 and VR2385 groups at 35 DPI (*p* = 0.0136). At 43 DPI, the NA titers of the chimeric virus groups were significantly higher than that of VR2385 group (*p* = 0.0382 and *p* = 0.046, respectively) (**Fig. 8D**).

### The GP4 or M Gene Shuffled Chimeric Viruses were Genetically Stable in Pigs

The GP4 or M chimeric viruses recovered from infected pigs at 14 DPI were sequenced for the respective shuffled gene regions. Sequence analyses showed that, with the exception of chimera MTS5, the shuffled target gene sequences of the seven other chimeric viruses recovered from infected pigs were identical to that of the original virus inocula. For the chimera MTS5, there was a “C” to “T” silent mutation at nt position 243 of the M gene. The results suggested that the chimeric viruses were genetically stable in pigs during acute infection.

### Amino Acid Mutations Introduced by DNA Shuffling in Chimeric Viruses may Contribute to the Altered Cross-neutralizing Capability of the Chimeras

**Figure 9:**
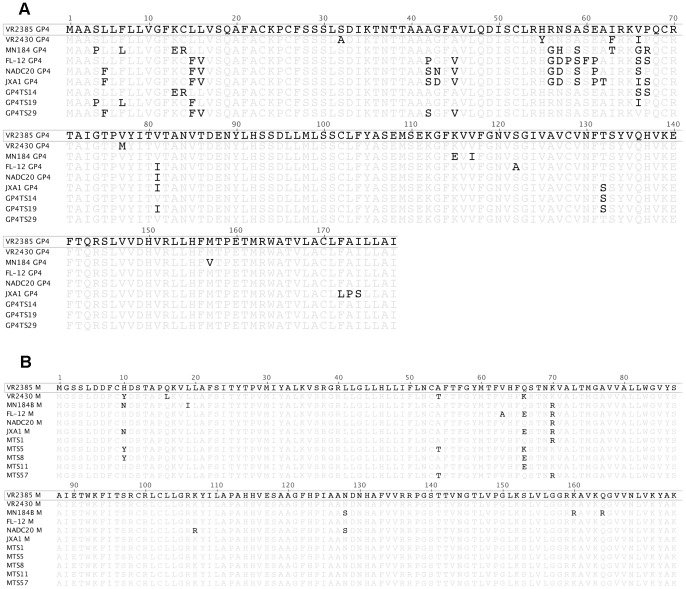
Sequence comparison and analyses of GP4 (Panel A) and M (Panel B) amino acid sequences of chimeric viruses and their parents. Multiple sequence alignments of GP4 and M amino acid sequences of six parental virus strains and shuffled chimeric viruses were performed. The sequence of the backbone virus VR2385 is shown on top, and only differences for other strains are indicated in the alignment.

To explore the potential correlation between amino acid mutation and altered cross-neutralizing activities, the amino acid sequences of the GP4 or M of the 8 chimeric viruses and six parental viruses were compared and analyzed (**Fig. 9**). The results showed that DNA shuffling introduced amino acid changes in all eight chimeric viruses, and all of the mutated amino acid residues were derived from parental strains, meaning that there was no new amino acid residue created by recombination. Most of the nucleotide changes are silent mutations with no change in amino acid sequence, particularly in shuffled M gene chimeras. The potential N-linked glycosylation sites of amino acid residues 37, 84, 120 and 130 in GP4 were conserved in all the parental and chimeric viruses. The limited knowledge on the neutralizing epitopes in GP4 and M genes of the type 2 PRRSV prevented from further investigation on the potential relationship between the epitope changes and cross-neutralizing activities. However, sequence analyses showed that the serine residue at position 66 in GP4 of chimera GP4TS14 was the only mutation that matched both FL-12 and NADC20, when it was compared to the backbone virus VR2385. Therefore, it is possible that the serine at position 66 in GP4 may contribute to the enhanced cross-neutralizing antibody-inducing capability of chimera GP4TS14 against both FL-12 and NADC20.

In the M gene, the amino acid region 151–174 which was reportedly immunoreactive, was also conserved in parental and chimeric virus strains, with the exception of MN184B. The region flanking the arginine residue at position 70 of the chimera MTS57 is same as that of the MN184B and NADC20, which might contribute to the altered cross-neutralizing antibody-inducing ability of the chimera MTS57. It remains to be further determined if these particular mutations in GP4 and M genes correlated with any neutralizing epitope that contributed to the improved cross-neutralizing antibody-inducing ability of the chimeric viruses GP4TS14 and MTS57 against the heterologous PRRSV strains.

## Discussion

PRRS is one of the most economically important global swine diseases [2]–[4], and PRRSV shows extensive genetic and antigenic diversity [21], [22]. The antigenic variation mitigates the effectiveness of virus recognition and subsequent immune responses. Although the utilization of PRRSV MLV is an effective method to provide immunological protection against homologous or genetically similar strains, the protection by the MLVs against the heterologous strains of PRRSV is limited [21], [22], [73]. Therefore, enhancing the heterologous protection is a key for the development of the next generation of vaccines against PRRSV [21], [74].

DNA shuffling is a powerful tool to increase the viral evolutional rate through random recombination and generate useful chimeras with desired properties such as improved cross-neutralizing antibody-inducing capability. This technology is especially useful when the viral determinants of protective immunity and the mechanism of protection are not fully understood, which would prevent from designing novel vaccines by directly transplantation of the desired epitope and immunogenic regions.

Our previous studies demonstrated that the DNA shuffling of GP5 or GP5-M genes could lead to attenuation of PRRSV [63]. The chimeric viruses with shuffled GP5 or GP5-M replicated at lower levels and formed smaller plaques *in vitro*, and showed significant reductions in viral RNA loads in sera and lungs, and decrease in gross and microscopic lung lesions in pigs. We also showed that DNA shuffling of GP3 gene produced a chimera GP3TS22 with a significantly higher level of cross-neutralizing antibodies in pigs against a heterologous strain PRRSV FL-12 [64]. Since other structural proteins of PRRSV such as GP4 and M have also been shown to induce neutralizing antibodies [43], [54], [75], and since the GP4 is known to interact with PRRSV receptor CD163 [45], [46], it is important to investigate whether DNA shuffling of GP4 and M gene can enhance the cross-neutralizing antibody-inducing capability of chimeric viruses.

In this present study, the GP4 or M gene of six different strains of PRRSV from different lineages or sublineages were shuffled by DNA shuffling. Eight well-shuffled chimeric viruses including three GP4-shuffled chimeras and five M-shuffled chimera were successfully rescued. We further demonstrated that, in MARC-145 cells, the replication levels of these eight chimeric viruses were similar to that of their backbone strain VR2385 virus, suggesting that the DNA shuffling of GP4 or M did not significantly impair the replication ability of these eight chimeric viruses *in vitro*. Similarly, in experimentally-infected pigs, the infection dynamics and replication levels of GP4 shuffled viruses were similar with that of backbone virus VR2385, although the serum viral titers of M gene-shuffled viruses MTS1, MTS5, MTS8 and MTS57 were significantly lower than that of backbone virus VR2385 at 7 DPI, but there was no difference observed at 14 DPI. The chimeric viruses elicited similar levels of anti-PRRSV antibody responses compared to the backbone VR2385 virus in pigs.

Most chimeric viruses still induce similar levels of neutralizing antibodies against the backbone virus VR2385, which is expected and consistent with our previous results from the shuffling of the GP3 genes [64]. Although the majority of the GP4 or M gene sequences in the chimeras are from other PRRSV strains, the sequences of the unshuffled regions in the chimeras are the same as that of the backbone strain VR2385, which also contain neutralizing epitopes, thus contributing to the induction of neutralizing antibodies against the backbone strain VR2385 or the genetically-similar strain VR2430.

Importantly, in this study we identified a chimera GP4TS14 with the shuffled GP4 gene that showed a significantly increased cross-neutralizing antibody-inducing capability against heterologous strains FL-12 and NADC20. Furthermore, the average neutralizing antibody titer in pigs infected by the chimera GP4TS14 against heterologous strains were also significantly higher than that induced by the backbone strain VR2385. Similarly, an M gene-shuffled chimera MTS57 was also found to induce a significantly higher level of cross-neutralizing antibody against heterologous strains MN184B and NADC20. Sequence analyses of the shuffled viruses revealed that the serine mutation at position 66 of GP4 and the arginine mutation at position 70 in M, both located at the ectodomains of GP4 and M [76], could potentially be related to the enhanced cross-neutralizing antibody-inducing capability of the chimeras against heterologous strains. However, a recent report argued that the major envelope protein surface epitopes were disassociated with the virus neutralization [77]. So it remains to be determined if these positions are located within or near the neutralizing epitopes, directly or indirectly influence the neutralizing antibody induction of chimeras.

The results of this study further demonstrate that the DNA shuffling of PRRSV structural proteins can be used to enhance the capability of chimeric viruses to induce broad cross-neutralizing antibody against heterologous strains. It is well known that the neutralizing antibodies play a critical role in the immunological control of many viral infections, and thus are crucial for anti-PRRS immunity [78]. Passive transfer of anti-PRRSV neutralizing antibodies was shown to confer sterilizing immunity against PRRSV viremia [79]. Therefore, rationale design of chimeric viruses with enhanced cross-neutralizing antibody-inducing capability may lead to the eventual development of a broadly protective vaccine against PRRSV.
